# Two eARCHT3.0 Lines for Optogenetic Silencing of Dopaminergic and Serotonergic Neurons

**DOI:** 10.3389/fncir.2019.00004

**Published:** 2019-02-01

**Authors:** Alexandra Krol, Violeta G. Lopez-Huerta, Taylor E. C. Corey, Karl Deisseroth, Jonathan T. Ting, Guoping Feng

**Affiliations:** ^1^McGovern Institute for Brain Research, Department of Brain and Cognitive Sciences, Massachusetts Institute of Technology, Cambridge, MA, United States; ^2^Stanley Center for Psychiatric Research, Broad Institute of Massachusetts Institute of Technology (MIT) and Harvard, Cambridge, MA, United States; ^3^Institute of Cellular Physiology, Department of Neurodevelopment and Physiology, National Autonomous University of Mexico, Mexico City, Mexico; ^4^Department of Bioengineering, Stanford University, Stanford, CA, United States; ^5^Department of Psychiatry and Behavioral Sciences, Stanford University, Stanford, CA, United States; ^6^Howard Hughes Medical Institute, Stanford University, Stanford, CA, United States; ^7^Human Cell Types, Allen Institute for Brain Science, Seattle, WA, United States

**Keywords:** opsin, inhibitory, serotonergic, dopaminergic, mouse, eArchT3.0

## Abstract

Dopaminergic and serotonergic neurons modulate and control processes ranging from reward signaling to regulation of motor outputs. Further, dysfunction of these neurons is involved in both degenerative and psychiatric disorders. Elucidating the roles of these neurons has been greatly facilitated by bacterial artificial chromosome (BAC) transgenic mouse lines expressing channelrhodopsin to readily enable cell-type specific activation. However, corresponding lines to silence these monoaminergic neurons have been lacking. We have generated two BAC transgenic mouse lines expressing the outward proton pump, enhanced ArchT3.0 (eArchT3.0), and GFP under control of the regulatory elements of either the dopamine transporter (DAT; Jax# 031663) or the tryptophan hydroxylase 2 (TPH2; Jax# 031662) gene locus. We demonstrate highly faithful and specific expression of these lines in dopaminergic and serotonergic neurons respectively. Additionally we validate effective and sensitive eArchT3.0-mediated silencing of these neurons using slice electrophysiology as well as with a well-established behavioral assay. These new transgenic tools will help expedite the study of dopaminergic and serotonergic system function in normal behavior and disease.

## Introduction

Neuroscience research has been revolutionized by cell-type specific access to neurons, by optical control of neuronal firing, and particularly by the intersection of the two. Since initial reports of optically gated ion channels and pumps, many new varieties and variants with increased sensitivity, kinetics, and various improved properties have been generated (Fenno et al., 2011; Mattis et al., 2011). Previously we generated a suite of cell type-specific bacterial artificial chromosome (BAC) transgenic lines to drive channelrhodopsin-2 (ChR2) expression in parvalbumin, serotonergic, cholinergic and GABAergic neurons (JAX# 012355, 014555, 014548, 014545, 014546; Zhao et al., 2011), which have been used widely in the field (Herde et al., 2013; Nelson et al., 2014; Van Dort et al., 2015; Li et al., 2016b; Latchoumane et al., 2017; Zhang et al., 2018). However, a complementary suite of silencing lines has been lacking. Here, we present two mouse lines expressing enhanced ArchT3.0 (eArchT3.0) that allow efficient optogenetic silencing of dopaminergic and serotonergic cells. By brightly co-expressing GFP, these lines are also useful for visualization of these neurons. ArchT is an outward proton pump from *Halorubrum sodomense* strain TP009 (Han et al., 2011). In eArchT3.0, the addition of endoplasmic reticulum export and neurite trafficking sequences produces enhanced membrane localization with correspondingly reduced intracellular accumulation, leading to subsequently much larger photocurrents. eArchT3.0 has a two-fold increase in photocurrent compared to unoptimized ArchT or Arch, measured *in vitro*, and double the current of the enhanced halorhodopsin chloride pump (eNpH3.0; Mattis et al., 2011).

Dopaminergic and serotonergic neurons control many diverse processes in the brain. Classically the main dopaminergic centers in the midbrain, the ventral tegmental area (VTA) and substantia nigra pars compacta (SNc), are mostly associated with reward processing in varied contexts (Wise, 2004). In particular, these neurons fire for reward prediction error (Schultz et al., 1997; Bayer and Glimcher, 2005; Cohen et al., 2015). In the clinic, dysregulation of dopamine leads to both motivational changes, for instance with drug abuse (Everitt and Robbins, 2005), and to movement disorders, such as difficulties initiating movement seen in Parkinson’s disease due to dopaminergic neuron degeneration in the sNC (Redgrave et al., 2010). The serotonergic system, in particular the raphe nuclei in the brainstem, the largest of which is the dorsal raphe nucleus (DRN), has also been associated with reward learning (Liu et al., 2014; Cohen et al., 2015). In the clinic, serotonin misregulation has been particularly implicated in depression, impulsivity, and anxiety in psychiatric disorders (Ansorge et al., 2004; Michelsen et al., 2007). Indeed, both dopaminergic and serotonergic signaling are key targets of some of the most widely used antipsychotic and antidepressant drugs (Li et al., 2016a; Yohn et al., 2017). However, our understanding of the functional roles of these circuits remains far from complete and a much more detailed and nuanced understanding is required to design better medications. The complexity and wide-ranging roles of these systems is beginning to be revealed using tools such as optogenetics which allow cell-type specific manipulations and fine temporal control. For example, the use of optogenetics has identified causal roles of VTA dopaminergic neurons in mediating depression symptoms from chronic stress (Tye et al., 2013), chronic defeat (Chaudhury et al., 2013), social interaction reward (Gunaydin et al., 2014) and dissected the dynamics of prediction error learning (Steinberg et al., 2013). For the serotonergic system, optogenetics has helped elaborate a complex role in reward-related behavior on multiple time scales (Liu et al., 2014; Miyazaki et al., 2014; Cohen et al., 2015) as well as diverse roles from signal processing in the olfactory system (Kapoor et al., 2016; Lottem et al., 2016) to regulating respiration and body temperature (Ray et al., 2011).

Here, we present two mouse lines with sensitive eArchT3.0-mediated silencing and high fidelity of expression in dopaminergic and serotonergic neurons. We characterize the specificity of the lines and their electrophysiology and validate the effectiveness of one of the lines in a well-established behavioral assay.

## Materials and Methods

### Vector Construction

To construct the targeting vectors, a pBlueScript-derived vector iTV1-WPRE was used to generate BAC targeting vectors, as previously described (Ting and Feng, 2014). BAC specific homology arms [dopamine transporter (DAT) box A and B or tryptophan hydroxylase 2 (TPH2) box A and B] were PCR amplified from template BAC DNA and cloned into iTV1-WPRE. In a separate mammalian expression vector, eArchT3.0 [ArchT (Han et al., 2011) but with a trafficking sequence and ER export motif added to the C-terminus similar to Mattis et al. (2011)] and enhanced green fluorescent protein cDNAs were combined using the P2A linker. We selected P2A with a C-terminal GSG linker because this combination was shown to be the most efficiently processed of all viral 2A elements (Kim et al., 2011) and thus was the most likely to enable a complete physical uncoupling of the opsin and fluorophore with no significant unprocessed protein fraction. Verification of proper expression and functionality were performed by transient transfection of the eArchT3.0-P2A-EGFP plasmid DNA into HEK293 cells and primary rat hippocampal neurons, followed by epifluorescent imaging and patch clamp recording. The eArchT3.0-P2A-EGFP cassette was then sub-cloned into the multiple cloning site of iTV1-A/B-WPRE to complete the BAC targeting vectors. In this final cloning step great care was taken to ensure minimal disruptions to the junction between 5′ homology arm and the start of the eArchT3.0 sequence, except for addition of a Kozak consensus sequence. BAC recombineering steps, including trimming of the DAT BAC clone, have been described previously (Ting and Feng, 2014).

### Animals

All experiments were done in accordance with NIH guidelines and approved by the MIT Institutional Animal Care and Use Committee. Mice were housed with a standard 12 h light/12 h-dark cycle (lights on at 07:00, lights off at 19:00) and given food and water *ad libitum*. eArchT3.0+ and eArchT3.0− mice of both genders from the same litters were used for experiments. Only heterozygous eArchT3.0+ mice were used for all experiments.

### Surgery

Five- to six-month-old mice were anesthetized with isoflurane and placed in a small animal stereotax (David Kopf Instruments, St. Tujunga, CA, USA). They were implanted with a manually-constructed optic fiber [200 um core, NA = 0.22 (FG200UCC, Thorlabs, Newton, NJ, USA)] held in a ceramic ferrule (CF270-10, Thorlabs, Newton, NJ, USA). Measurements were made relative to Bregma, coordinates: −3.1, 0.35–0.37, −4.3. The fiber was held in place to the skull with adhesive cement (C&B Metabond; Parkell Inc., Brentwood, NY, USA). The incision was closed with vetbond and mice were recovered on a heat pad in a clean cage. Mice were group housed pre and post surgery. Fiber placement was confirmed after behavior with serial coronal sections, anti-TH staining to identify the VTA and epifluorescent imaging (Supplementary Figure S1).

### Conditioned and Real-Time Place Aversion

Behavioral testing was performed 3.5 weeks post-surgery, allowing time for recovery. Mice were tested between 7 am and 7 pm during their light cycle. Pre-testing, mice were habituated at least 1 h in the behavioral testing room. For testing, single mice were place in a clear plexiglass open top chamber with equally sized left and right chambers (22 × 22 cm) and a smaller central chamber (9 × 22 cm) separated by dividers leaving a gap (9 cm) between the center chamber and the side chambers. Each trial began with placement of the mouse in a corner of the central chamber. Between each trial, the chamber was cleaned with 70% ethanol. On the 1st day mice were allowed to freely explore the chamber for 15 min with the light turned off. On the 2nd and 3rd days mice were allowed to freely move between the compartments for 30 min. Entry into one of the side chambers was paired with photostimulation (532 nm light, GL532T3-100 mW Shanghai Laser and Optics Century Co.). Laser power was approximately 5 mW at tip of fiber, but substantial ramping up of the laser seen upon turn on could have led to approximately ±1 mW during stimulation. To avoid overheating, optical stimulation was paused for 30 s if the mouse stayed in the stimulated zone over 30 s. The stimulated side was counterbalanced between animals and genotypes but kept constant for each individual animal between days. On the 4th day mice were allowed, as on the 1st day, to explore freely for 15 min with the laser turned off. Mouse movement was tracked and paired with photostimulation control using a video camera and EthoVision software (EthoVision XT, Noldus, Wageningen, Netherlands) through a Master-9 Pulse Stimulator (A.M.P.I, Jerusalem, Israel). For analysis, tracks were edited for the correct start and end times blind to condition. Dwell time in each predefined area was calculated automatically using EthoVision software.

### Immunohistochemistry and Image Quantification

Mice were deeply anesthetized with isoflurane, transcardially perfused with ice-cold PBS solution followed by fresh ice-cold 4% paraformaldehyde (PFA) in PBS. Brains were dissected out and postfixed overnight with 4% PFA at 4°C, and stored in PBS at 4°C. Sixty to hundred microgram sections were cut on a vibratome (Leica VT100S). Floating brain slices were permeabilized with 1.2% Triton X-100 in PBS for 15 min, washed 3 × 5 min in PBS and blocked 1 h in 5% normal goat serum, 2% BSA, 0.2% Triton X-100 in PBS (blocking buffer) for 1 h at room temperature. Sections were incubated with primary antibodies in blocking solution 1–3 overnights at 4°C. Slices were then washed three times with PBS for 20 min, incubated with secondary antibodies for 1–2 h at room temperature, washed 1× with DAPI (1:10,000) in PBS for 20 min then 2× with PBS for 20 min. Slice were mounted with 50% glycerol in Tris Buffer. Primary antibodies used were ms anti-TPH2 (1:500, Sigma, St. Louis, MO, USA to678), ms IgG1 anti-TH (1:1,000, immunostar 22941), rb anti p2A (1:1,000 Millipore, Burlington, MA, USA ABS31), and rat anti-DAT (1:1,000 Millipore, Burlington, MA, USA MAB369). Widefield epifluorescent images were capture using a 4× lens on a Olympus BX61 microscope with a PRIOR ProScan III motorized stage (Prior Scientific Instruments, UK) and CellSens Dimension 1.11 stitching software (Olympus). To quantify overlap, images were captured using Olympus Fluoview FV1000 confocal microscope, 20× lens. Three mice were imaged per condition, three sections per mouse. DAT sections, since they were coronal, were imaged bilaterally. Cells were counted manually using the ImageJ Cell Counter plugin (Schindelin et al., 2012). To quantify cell number and TPH2 and DAT expression levels in old animals over 1 year old, serial 60 um sagittal sections were collected from TPH2 and DAT-eArchT3.0 BAC transgenic animals and immunostained with TPH2, DAT or TH. Every third section from three animals was confocal imaged as described above; sections were matched referenced to the midline. Cells were counted automatically with CellProfiler (Lamprecht et al., 2007) using primary object detection and the Otsu global thresholding method. For the DAT-eArchT3.0 transgenic line, DAT immunostaining did not clearly distinguish individual dopaminergic cell bodies, so TH staining was used. To quantify DAT expression levels, DAT staining in the striatum was used to take advantage of relatively uniform coverage by dopaminergic terminals. To correct for absence of terminals around nuclei and fibers of passage, a mask was used to remove those areas from quantification. For the TPH2 transgenic line, TPH2 immunostaining was used both to quantify cell number and to assess expression levels. To correct for variable cell number between sections and animals, intensity levels were only measured from detected cells and normalized to total cell area per image.

### Slice Preparation for Electrophysiological Recordings

Sagittal or coronal slices (250 μm) were obtained from DAT-eArchT3.0 or TPH2- eArchT3.0 expressing mice at 30–40 p.n. days. Mice were anesthetized *via* isoflurane inhalation and perfused transcardially using cold saline containing (in mM): 194 sucrose, 30 NaCl, 4.5 KCl, 1.2 NaH_2_PO_4_, 0.2 CaCl_2_, 2 MgCl_2_, 26 NaHCO_3_, and 10 D-(+)-glucose saturated with 95% O_2_ and 5% CO_2_, pH = 7.4, 320–340 mOsm/L. Slices were cut using a slicer (VT1200 S, Leica Microsystems In., Grove, IL, USA) and then incubated for 10–15 min in a holding chamber (BSK4, Scientific System Design Inc., Little Ferry, NJ, USA) at 32°C with regular artificial cerebral spinal fluid containing the following in mM: 136 NaCl, 3.5 KCl, 1 MgCl_2_, 2.5 CaCl_2_, 26 NaHCO_3_ and 11 glucose saturated with 95% O_2_ and 5% CO_2_, followed by at least 1 h recovery at room temperature (21–25°C) before recording.

### Electrophysiological Recordings

We performed whole cell patch-clamp recordings with borosilicate glass pipettes (KG33, King Precision Glass) heat polished to obtain direct current resistances of ~4–6 MΩ. Pipettes were filled with an internal solution containing in mM: 120 K-Gluconate, 2 MgCl_2_, 10 HEPES, 0.5 EGTA, 0.2 Na_2_ATP, and 0.2 Na_3_GTP. The recordings were made with a microelectrode amplifier with bridge and voltage clamp modes of operation (Multiclamp 700B, Molecular Devices, San Jose, CA, USA). Cell membrane potential was held at −60 mV, unless specified otherwise. Signals were low-pass filtered at 2 kHz and sampled at 10–20 kHz with a Digidata 1440A (Molecular Devices, San Jose, CA, USA), and data were stored on a computer for subsequent off-line analysis. Cells in which the series resistance (Rs, typically 8–12 MΩ) changed by >20% were excluded for data analysis. In addition, cells with Rs more than 20 MΩ at any time during the recordings were discarded. In some cases conventional characterization of neurons was made in voltage and current clamp configurations.

DAT+ or TPH+ neurons were identified for recordings on the basis of GFP expression visualized with a microscope equipped with a GFP filter (BX-51WI, Olympus). We also targeted GFP negative (−) neurons to confirm the specificity of eArchT3.0 effects in neurons. Photo stimulation parameters were 532 nm and 1–4 mW per mm^2^ (GL532T3-100 mW Shanghai Laser and Optics Century Co.). Neurons were held at −70 mV during photocurrent measurements. To confirm the ability of photo stimulation to inhibit action potential firing, action potentials were induced by continuous positive current injection until tonic firing was reached.

### Statistics

All data were transferred to GraphPad Prism for analysis and graphing. Electrophysiological data are presented as mean ± SEM, and with the values given for each experiment referring to the number of cells analyzed unless noted otherwise. All error bars indicate SEM. The significance level for all tests was *p* < 0.05. Group results were compared by using an unpaired Student’s *t*-test. Quantification of cell immunohistochemistry results are presented as mean ± SD. Cell number quantification in old animals is presented as a scatter plot showing the number of cells across three sections per animal with mean ± SD overlaid. For immunofluorescent intensity the median intensity of each section from three animals is shown and for TPH2 staining the intensity is normalized to the cell area coverage in each section. Immunohistochemistry results were compared using an unpaired Student’s *t*-test; the significance level for all tests was *p* < 0.05. Behavioral testing data is presented as a scatter plot with individual animal’s performance with mean and SEM error bars overlaid. Results were compared using one-way analyses of variance (ANOVA) and adjusted *P* values were calculated using Sidak’s multiple comparison.

## Results

### Generation of eArchT3.0 BAC Transgenic Lines

Two eArchT3.0 lines were generated, the first to silence dopaminergic neurons and the second to silence serotonergic neurons using an optimized archeorhodopsin, eArchT3.0 (Han et al., 2011; Mattis et al., 2011). Both lines were generated using an improved BAC-transgenic approach that avoids overexpression of endogenous genes (Ting and Feng, 2014). In both cases, the eArchT3.0 cDNA was inserted into the BAC clone followed by the self-cleaving peptide p2A and GFP. This allows both bright visualization of expressing neurons, including in fresh tissue, through cytoplasmic GFP expression as well as localization of the Arch protein by immunochemical detection of the P2A fragment remaining at the eArchT3.0 C-terminus. To improve RNA stability, and hence protein expression levels, a woodchuck hepatitis virus posttranscriptional regulatory element (WPRE) and a bovine growth hormone polyadenylation signal (BGHpA) were added. For dopaminergic expression, this engineered cDNA was placed after the translation initiation codon, ATG, of the DAT gene in a BAC clone. DAT is present in dopaminergic neurons to reuptake dopamine from the synaptic cleft to be repackaged into synaptic vesicles and is selective for dopaminergic neurons (Augood et al., 1993; Lammel et al., 2015). For serotonergic expression, a TPH2 BAC clone was used. TPH2 is an enzyme that catalyzes the first and rate limiting step in serotonin production and is specific for serotonergic neurons (Walther et al., 2003).

### eArchT3.0 Expression in Dopaminergic Neurons of DAT-eArchT3.0 BAC Transgenic Mice

**Figure 1:**
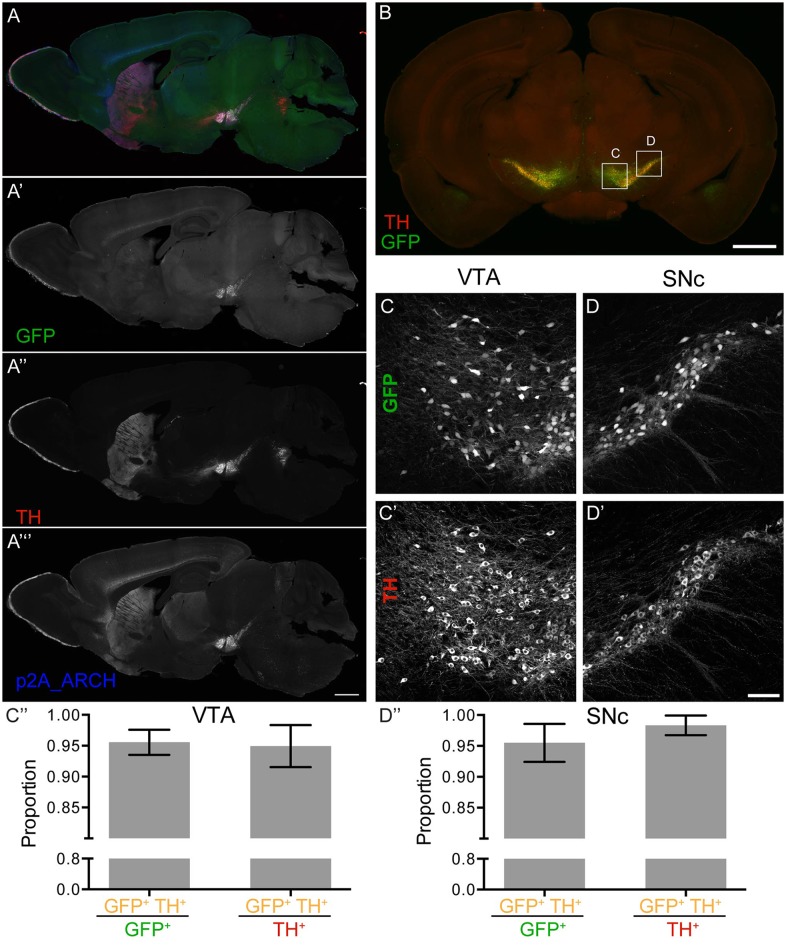
Dopamine transporter (DAT)-enhanced ArchT3.0 (eArchT3.0) expression pattern. **(A)** Merged image **(A′–A″′)** of a sagittal section highlighting the ventral tegmental area (VTA), substantia nigra pars compacta (SNc) and striatum. **(A′)** Native GFP expression labeling targeted neurons. **(A″)** Tyrosine hydroxylase (TH) staining labeling dopaminergic neurons. (**A″′)** P2A staining labeling P2A-tagged- eArchT3.0 protein, localizing both to cell bodies in the VTA and SNc, and neuronal terminals in the striatum. Scale bar 1 mm. **(B)** Example section used for quantification of overlap between TH and GFP. Scale bar 1 mm. **(C–C″)** Quantification in the VTA of GFP and TH overlap. **(C–C′)** Example image used for quantification. **(C)** GFP fluorescence. **(C′)** TH staining. **(C″)** Specificity of GFP expression (left hand side): the proportion of cells labeled for both TH and GFP out of all GFP positive cells (96 ± 2%). Coverage of TH positive cells by GFP expression (right hand side): the proportion of cells labeled for both TH and GFP out of all TH positive cells (95 ± 3%). **(D–D″)** Quantification in the SNc of GFP and TH overlap. **(D–D′)** Example images used for quantification. **(D)** GFP fluorescence. **(D′)** TH staining. Scale bar 100 um. **(D″)** Specificity of GFP expression (left hand side): the proportion of cells labeled for both TH and GFP out of all GFP positive cells (96 ± 3%). Coverage of TH positive cells by GFP expression (right hand side): the proportion of cells labeled for both TH and GFP out of all TH positive cells (98 ± 2%; quantification: mean ± SD, bilateral images from eight sections from three animals).

Accurate expression of GFP and eArchT3.0 protein in dopaminergic neurons was found in neurons of the VTA and SNc with excellent overlap between tyrosine hydroxylase (TH) staining, the rate-limiting enzyme for dopamine synthesis, and GFP expression (Figure 1). Quantification shows very high overlap between GFP and TH with both high specificity of GFP expression, most GFP positive cells were also TH positive (Figure 1C; 96 ± 2%, VTA, Figure 1D; 96 ± 3%, SNc) and high coverage of GFP expression, most TH positive cells were also GFP positive (Figure 1C; 95 ± 3% VTA, Figure 1D; 98 ± 2% SNc). These neurons were also DAT positive (Supplementary Figure S2). Although TH is specific for dopaminergic neurons of the midbrain, because TH catalyzes the first step in catecholamine biosynthesis, TH is also expressed in some neurons that are not dopaminergic or DAT positive, such as the catecholamine neurons of the locus coeruleus (Augood et al., 1993; Lorang et al., 1994). Highlighting the specificity of this line, the locus coeruleus (Figure 1A″) though labeled by TH is not correspondingly labeled by GFP (Figure 1A′). GFP expression was also present in dopaminergic neurons of the olfactory bulbs (Pignatelli and Belluzzi, 2017; Supplementary Figure S2) as well as small TH-positive interneurons in the striatum (Ibáñez-Sandoval et al., 2010; Xenias et al., 2015; Supplementary Figure S3F). Notably, the GFP signal was bright enough to be seen and quantified without enhancement. This indicated a high level of eArchT3.0 expression since production of the two was stoichiometric at the mRNA level and likely protein level. Bright GFP expression also makes these mice a useful tool for targeting dopaminergic neurons in slice recordings. To determine possible effects of long-term eArchT3.0 expression or the transgenic approach on dopaminergic neuronal number or DAT gene expression levels, cell number in the midbrain (Supplementary Figures S4A,D) and DAT intensity levels in the striatum (Supplementary Figures S4B,E) in old animals over 1 year old were quantified. There was no difference in cell number (689 ± 201, 793 ± 189, n.s. unpaired *t*-test, *n* = 3 animals, Supplementary Figure S4D) or protein levels (0.014 ± 0.006, 0.015 ± 0.004, n.s. unpaired *t*-test, *n* = 12 sections, Supplementary Figure S4E) between the controls and DAT-eArchT3.0 animals.

We observed some off-target expression of eArchT3.0-GFP in subplate neurons and subiculum (Supplementary Figure S3F). A low level of expression was also seen sparsely in cells of the cortex (Supplementary Figure S3E). However, these neurons are spatially distinct from the VTA and SNc. Thus, separable control of these two populations should be easily achieved with optic fiber placement.

### eArchT3.0 Expression in Serotonergic Neurons of TPH2-ArchT3.0 BAC Transgenic Mice

**Figure 2:**
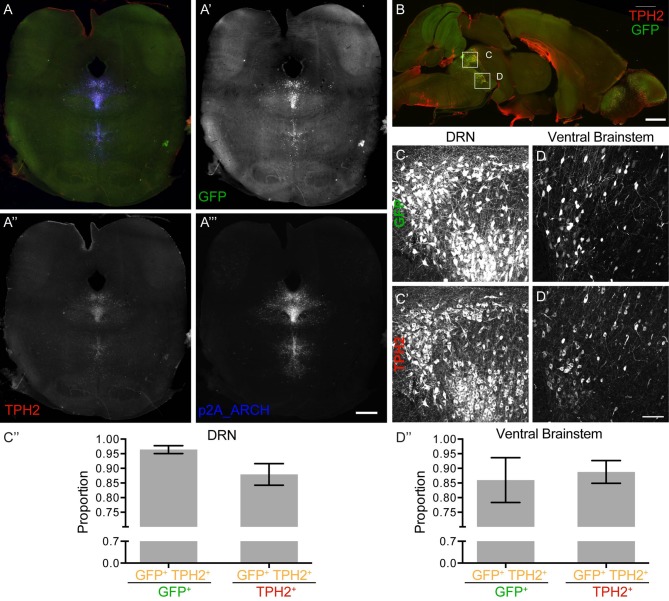
Tryptophan hydroxylase 2 (TPH2)- eArchT3.0 expression pattern. **(A)** Merged image **(A′–A″′)** of a coronal section, highlighting the dorsal raphe nucleus (DRN). **(A′)** Native GFP expression labeling targeted neurons. **(A″)** TPH2 staining labeling serotonergic neurons. **(A″′)** P2A staining labeling P2A-tagged-eArchT3.0 protein. Scale bar 1 mm. **(B)** Example section used for quantification of overlap between TPH2 and GFP. Scale bar 1 mm. **(C–C″)** Quantification in the dorsal raphe nucleus (DRN) of GFP and TPH2 overlap. **(C–C′)** Example image used for quantification. **(C)** GFP fluorescence. **(C′)** TPH2 staining. **(C″)** Specificity of GFP expression (left hand side): the proportion of cells labeled for both TPH2 and GFP out of all GFP positive cells (96 ± 1%). Coverage of TPH2 positive cells by GFP expression (right hand side): the proportion of cells labeled for both TPH2 and GFP out of all TPH2 positive cells (88 ± 4%). (**D–D″)** Quantification in the ventral brainstem of GFP and TH overlap. **(D–D′)** Example image used for quantification. **(D)** GFP fluorescence. **(D′)** TH staining. Scale bar 100 um. **(D″)** Specificity of GFP expression (left hand side): the proportion of cells labeled for both TPH2 and GFP out of all GFP positive cells (86 ± 8%). Coverage of TPH2 positive cells by GFP expression (right hand side): the proportion of cells labeled for both TPH2 and GFP out of all TPH2 positive cells (89 ± 4%; quantification: mean ± SD, bilateral images from eight sections from three animals).

Serotonergic neurons in both the DRN and the ventral brainstem, which contains multiple more sparsely distributed serotonergic nuclei, all faithfully expressed eArchT3.0-GFP (Figure 2). Expression of GFP was as bright as in the DAT-eArchT3.0 line as was the excellent fidelity of expression. Quantification confirmed high overlap between GFP expression and TPH2; assessing specificity of GFP expression determined most GFP positive cells were also TPH2 positive (Figure 2C, 96 ± 1% in DRN, Figure 2D, 86 ± 8%, ventral brainstem) and assessing coverage of GFP expression determined most TPH2 positive cells were GFP positive (88 ± 4% in DRN, Figure 2D, 89 ± 4% ventral brainstem). TPH2 immunostaining was sometimes faint so overlap may be underestimated. Altogether this indicates faithful and robust expression of eArchT3.0 in serotonergic neurons in the brain. As for the DAT line, we determined possible long-term effects of the transgene in old animals over 1 year old by quantifying serotonergic neuronal number and TPH2 intensity levels in the DRN (Supplementary Figures S4C,F,G). There was no difference in cell number (1,032 ± 159, 728 ± 224, n.s. unpaired *t*-test, *n* = 3 animals, Supplementary Figure S4F) or protein levels (0.006 ± 0.005, 0.004 ± 0.004, n.s. unpaired *t*-test, *n* = 9 sections, Supplementary Figure S4G) between the controls and TPH2-eArchT3.0 animals.

Off-target expression of eArchT3.0-GFP at a lower level of expression was present in granule cells of the cerebellum (Supplementary Figures S5A′,B′), as well as sparse scattered cells in the cortex (Supplementary Figures S5A″′,B″′).

### Electrophysiological Characterization of eArchT3.0-Expressing Dopaminergic Neurons

**Figure 3:**
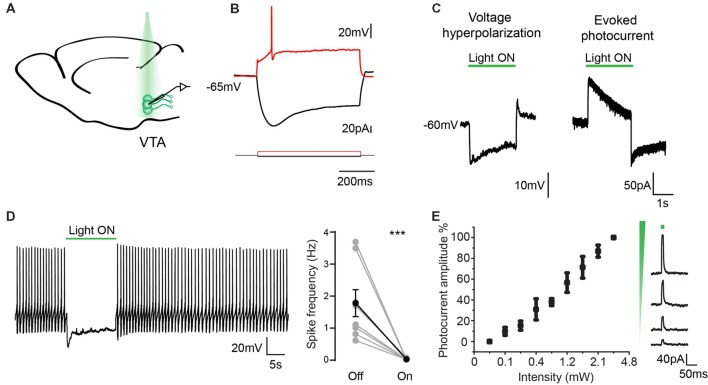
Electrophysiological characterization of eArchT3.0 in dopaminergic neurons. **(A)** Schematic representation of the recording and stimulation preparation. **(B)** Typical VTA neuron response to hyperpolarizing and depolarizing current steps. **(C)** Whole cell recording in current (left) and voltage clamp (right) of the photoresponse in an example eArchT3.0-GFP expressing neuron from DAT-eArchT3.0 transgenic mouse (green bar, 532 nm light). **(D)** Sample trace of light-induced inhibition of spikes generated by current injection (left). During 10-s of photo stimulation (green bar), action potentials are suppressed. Population summary (right). Total spike rate before and during 10 s of photo stimulation (*n* = 10 neurons, mean ± SEM paired two-tailed *t*-test, ***p = 0.01, all cells showed inhibition). **(E)** Intensity response curve, data represents mean ± SEM (*n* = 8, left). Sample traces for evoked photocurrent at different light intensities (right).

To analyze the ability of genetically encoded eArchT3.0 to enable reversible silencing of neuronal activity, we recorded from GFP-positive neurons in the VTA (*n* = 10, from five mice; Figure 3A). These neurons had characteristic properties of TH cells including: presence of a depolarized resting membrane potential (RMP; −52 ± 8 mV), wide action potentials (1.7 ± 0.16 ms half width) and Ih current (sag amplitude 34.9 ± 5.2 mV; Figure 3B; Chieng et al., 2011; Krashia et al., 2017). We demonstrated that GFP-positive neurons were sensitive to 532 nm light which led to eArchT3.0 mediated-hyperpolarization and evoked photocurrent with similar kinetics as reported previously (Figures 3C–E; Han et al., 2011; Mattis et al., 2011). A roughly linear relationship between photocurrent and light intensity was found as shown by the input-output curve (Figure 3E). At −45 ± 4.1 mV neurons had spontaneous tonic firing around 2.5 ± 0.9 Hz and application of 532 nm light (1–4 mW) for 10 s was highly effective at silencing firing precisely during light exposure (Figure 3D, paired *t*-test, *p* = 0.01). We repeated this manipulation 3–5 times on each neuron recorded and all cells showed inhibition. Lastly, to confirm the specificity of GFP-eArchT3.0 expression we also targeted GFP negative (−) cells in the VTA (Supplementary Figures S6A–C). We did not observe any evoked photocurrent in neurons recorded in VTA, or any significant changes in the RMP (light OFF −52.4 ± 2.2 mV and light ON −53.2 ± 2.1 mV, n.s. unpaired *t*-test, *n* = 9) or firing rate when there was spontaneous spiking (light OFF 5.9 ± 2 Hz and light ON 6.1 ± 2.2, n.s. unpaired *t*-test, *n* = 5; Supplementary Figure S6C). These results give us confidence that neurons that do not express GFP do not express eArchT3.0 and they are not affected by photo stimulation.

### Electrophysiological Characterization of eArchT3.0-Expressing Serotonergic Neurons

**Figure 4:**
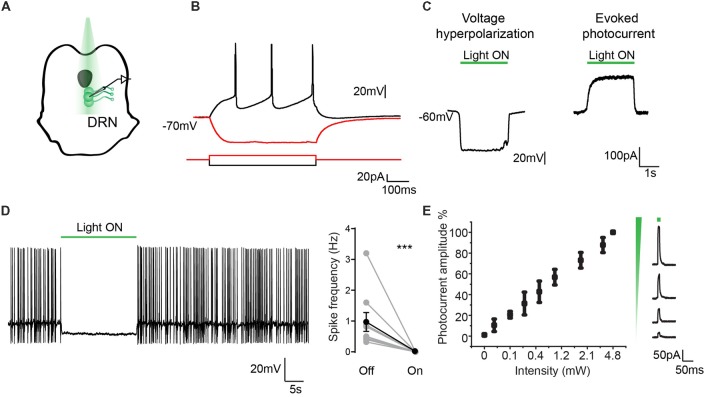
Electrophysiological characterization of eArchT3.0 in serotonergic neurons. **(A)** Schematic representation of the recording and stimulation preparation. **(B)** Typical DRN neuron response to hyperpolarizing and depolarizing current steps. **(C)** Whole cell recording in current (left) and voltage clamp (right) of the photoresponse in an example eArchT3.0-GFP expressing neuron from TPH2- eArchT3.0 transgenic mouse (green bar, 532 nm light). **(D)** Sample trace of light-induced inhibition of spikes generated by current injection (left). During 10-s of photostimulation (green bar), action potentials are suppressed. Population summary (right). Total spike rate before and during 10 s of photostimulation. (*n* = 12 neurons, mean ± SEM paired two-tailed *t*-test, ***p = 0.014, all cells showed inhibition). **(E)** Intensity response curve, data represents mean ± SEM (*n* = 9, left). Sample traces for evoked photocurrent at different light intensities (right).

The same characterization was carried out in DRN neurons. Recordings from GFP-positive cells (*n* = 11, from four mice) showed typical characteristics of serotonergic neurons (Figure 4A) with a RMP of −59 ± 2.5 mV and action potential threshold of −40.5 ± 0.9 mV (Figure 4B; Mlinar et al., 2016; Asaoka et al., 2017). As reported before for eArchT3.0 activation and found in the dopaminergic neurons, 532 nm light led to neuron hyperpolarization and sustained evoked photocurrent (Figure 4C; Han et al., 2011; Mattis et al., 2011). Similarly, a linear relationship between photocurrent and light intensity was found (Figure 4E). At −45 mV neurons had spontaneous tonic or irregular firing patterns at 0.96 ± 0.3 Hz and application of 532 nm light (1–4 mW) for 10 s was highly effective at silencing neuronal activity (Figure 4D, paired *t*-test, *p* = 0.014). We repeated this manipulation 3–5 times on each neuron recorded and all cells showed inhibition. Again, to confirm the specificity of GFP-eArchT3.0 expression we also targeted GFP (−) cells in the DRN (Supplementary Figures S6D–F). We observed that GFP (−) neurons did not show any current when stimulated with light and only showed a slight change in the firing frequency (light OFF 0.85 ± 0.26 Hz and light ON 0.81 ± 0.35 Hz, n.s. unpaired *t*-test, *n* = 8) likely due to changes in the surrounding inhibited neurons (Supplementary Figure S6F). These results give us confidence that neurons that do not express GFP do not express eArchT3.0 and they are not affected by photo stimulation.

### Optogenetic Activation of eArchT3.0 in VTA Is Sufficient to Drive Conditioned Place Aversion

Our slice experiments determined that eArchT3.0 expression was effective at silencing dopaminergic and serotonergic neurons. To determine whether eArchT3.0 was effective in a behavioral assay we used the dopaminergic eArchT3.0 line. Inhibition of dopaminergic neurons has been shown to be sufficient to mediate place aversion (Tan et al., 2012; Danjo et al., 2014). In place aversion, a location in an arena is paired with either an aversive condition, such as a foot shock, or a neuronal signal, such as inhibition of the VTA, over several conditioning days. In conditioned place aversion, aversive conditioning is delivered to an animal restrained to the location. In real-time place aversion, aversive conditioning is delivered to a freely exploring animal upon entry into the location. In both assays, animals avoid the aversive conditioned-location in a post-conditioning test day. In real-time place aversion, a decreased dwell time in the conditioned location can also be observed during conditioning days. Both conditioned place aversion and real-time place aversion have been reported previously upon inhibition of dopaminergic neurons *via* optogenetic activation of local inhibitory neurons in the VTA (Tan et al., 2012) or by direct optogenetic inactivation of the VTA with viral-driven expression of eNpH3.0 (Tan et al., 2012) or eArchT3.0 in a TH Cre mouse (Danjo et al., 2014).

**Figure 5:**
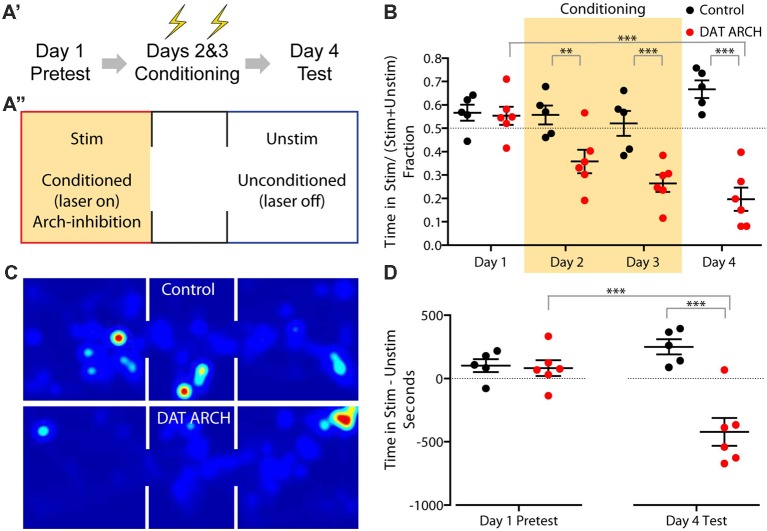
Place aversion induced by DAT-eArchT3.0 mediated VTA inhibition. **(A)** Experimental timeline **(A′)** and schematic of arena **(A″)**. Mice were stimulated unilaterally when in the stimulated (stim) zone during conditioning days. **(B)** Fraction of time spent in the stimulated zone on each day. **(C)** Heatmap of time spent in each area (schematized above) on day 4 by example control or DAT-eArchT3.0 animal. **(D)** The difference in time spent in the stimulated zone vs. the unstimulated zone before and after conditioning. Data are plotted mean ± SEM, *n* = 5–6. Analysis uses one-way analyses of variance (ANOVA) and reports Sidak’s multiple comparison adjusted *P* values, **P < 0.01, ***P < 0.001.

To validate our DAT eArchT3.0 line in a similar assay, we used a three chamber arena with two equivalent larger chambers connected by a smaller third one (Figure 5A”), allowing mice to explore freely between the chambers, performing the assay over 4 days (Figure 5A’). On the first, pretest, day mice were allowed to explore with the laser turned off. On the next two, conditioning, days, the laser was triggered when mice entered the designated conditioning side, leading to unilateral eArchT3.0-mediated inhibition of the VTA. On the 4th day, mice were tested as on day one without light delivery. As expected, on the 1st day at pretest, mice showed no preference for one chamber vs. the other (Figures 5B,D). Following conditioning using eArchT3.0-mediated inhibition of DAT^+^ VTA neurons, aversion to the conditioned side was seen upon the fourth test day (Figures 5B,D). Mice spent significantly less time in the conditioned side (Figure 5C) both in the fraction of time spent (Figure 5B) and in net time (Figure 5D). Real-time place aversion during training was also observed with less time spent in the conditioned area each conditioning day (Figure 5B). These changes were not seen for the control, wildtype, littermate mice, which were implanted and stimulated the same way but did not express eArchT3.0. Overall this demonstrates that the DAT-eArchT3.0 line is a sensitive tool for effectively inducing inhibition of dopaminergic neurons *in vivo*.

## Discussion

We have generated and characterized two new lines of transgenic mice for optogenetically silencing dopaminergic or serotonergic neurons robustly and reproducibly. These two mouse lines will be shared with the community *via* Jackson Laboratory: DAT-eArchT3.0-GFP (Jax# 031663) and Tph2-eArchT3.0-GFP (Jax# 031662). Generating these lines using optimized trimmed BACs avoided unwanted overexpression of other genes in the BAC clones, a concern with previous lines such as Chat-ChR2 (Kolisnyk et al., 2013; Ting and Feng, 2014). Reassuringly quantification of cell number and DAT or TPH2 intensity levels in old animals showed little long-term effects in transgenic animals. Additionally, the characteristic electrophysiological properties of both dopaminergic and serotonergic neurons expressing eArchT3.0 were consistent with the published literature for non-eArchT3.0 expressing neurons. Both lines also showed good fidelity and coverage for dopaminergic and serotonergic neurons. The co-expressed GFP fluorescence is also clearly visible without immunostaining amplification and can be used both to target cells for *in vitro* recordings and for morphological studies, making these lines useful for a range of eArchT3.0-independent applications. Both lines show excellent and sensitive silencing *in vitro*, although it is worth noting that the same level of photostimulation may not completely bock firing *in vivo*, where neurons can display higher firing rates and have additional extrinsic drives. Nonetheless our robust behavioral results in the conditioned place aversion assay using the DAT line indicate a substantial reduction in neuronal activity.

Compared to the original ArchT pump (Han et al., 2011), eArchT3.0 has an added endoplasmic reticulum export sequence and neurite trafficking sequencing, enhancing localization of the pump at the membrane (Mattis et al., 2011). Further, in our lines, GFP is not fused to the pump and instead is produced ratiometrically through a P2A self-cleaving peptide. Compared to the other widely used pumps, eNpHR3.0 (Gradinaru et al., 2010), unmodified Arch (Chow et al., 2010) and unmodified ArchT (Han et al., 2011), eArchT3.0 has much greater photocurrent and this difference is greater with increased light power density (Mattis et al., 2011). Both enhanced Arch (eArch3.0) and enhanced ArchT (eArchT3.0) proteins pass significantly more current than the original variants and this difference increases as the light power density increases (Mattis et al., 2011). Of consideration for certain applications, the maximum wavelength sensitivity of eArchT3.0 is between 520–560 nm. This is green shifted compared to eNPHr3.0 which is 560–590 and Jaws, which is further red shifted, with more photocurrent at 632 nm compared to eNpHR3.0 (Gradinaru et al., 2010; Mattis et al., 2011; Chuong et al., 2014). With all of these pumps caution is warranted (Wiegert et al., 2017); all three for instance exhibit comparable post-illumination rebound spiking (Chuong et al., 2014; Mahn et al., 2016) which can be avoided using a gradual off-ramp of illumination (Chuong et al., 2014; Mahn et al., 2016). Additional challenges can arise from use of these inhibitors at axon terminals. In such small cellular compartments, ionic changes can be exacerbated. In particular, prolonged activation of terminals on the 2–5 minute timescale using eArchT3.0 can cause significant increase in spontaneous activity, due to alkalinization of the boutons and the subsequence pH-dependent increase in intracellular calcium (Mahn et al., 2016; Wiegert et al., 2017).

The use of transgenic lines for targeted opsin expression presents several advantages compared to expressing opsins virally in combination with a Cre-line. Unlike virally mediated opsin expression, these lines will have less variable levels of expression from mouse to mouse which can result from variable viral injection and neuronal uptake and will eliminate the need to wait for viral expression post surgery. The ability to drive earlier eArchT3.0 expression could also facilitate future studies of the developmental roles of serotonergic and dopaminergic signaling. Admittedly, the need to implant fibers to optically silence the neurons may still limit developmental questions to acute experiments, up to an age when mice are not rapidly growing and when they are not still dependent on maternal care. Additionally, by dissociating eArchT3.0 expression from Cre expression, crossed in Cre lines can be used to target additional tools or neuronal populations of interest, for instance either for gene knockout or other opsin expression. By expressing eArchT3.0 in one cell population and channelrhodopsin in another, either using Cre-mediated recombination or a different transgenic line, different populations of neurons could be stimulated and inhibited in the same animal. Such a combination of tools could prove particularly powerful when exploring the effects of neuromodulatory neurons on other circuits on both the short and long timescales at which they act. We hope that these two new DAT and TPH2-eArchT3.0 lines will help facilitate a refined understanding of the roles of dopaminergic and serotonergic systems.

## Author Contributions

AK, JT and GF conceived the study and oversaw the project. AK screened the founder mouse lines for successful transgenics, performed the staining, imaging and quantification to characterize transgene expression as well as surgeries and place aversion behavior. VL-H performed the electrophysiology and analysis. TC helped screen the founder lines, performed quantification and mapped fiber placement. KD provided the original eArchT3.0 construct. JT created and tested the BAC constructs and generated the mice. AK and GF wrote the manuscript with input from all authors.

## Conflict of Interest Statement

The authors declare that the research was conducted in the absence of any commercial or financial relationships that could be construed as a potential conflict of interest.

## References

[B1] Ansorge M. S. Zhou M. Lira A. Hen R. Gingrich J. A. (2004). Early-life blockade of the 5-HT transporter alters emotional behavior in adult mice. Science 306, 879–881. 10.1126/science.1101678 15514160

[B2] Asaoka N. Nishitani N. Kinoshita H. Kawai H. Shibui N. Nagayasu K.. (2017). Chronic antidepressant potentiates spontaneous activity of dorsal raphe serotonergic neurons by decreasing GABA_B_ receptor-mediated inhibition of L-type calcium channels. Sci. Rep. 7:13609. 10.1038/s41598-017-13599-3 29051549 PMC5648823

[B3] Augood S. J. Westmore K. McKenna P. J. Emson P. C. (1993). Co-expression of dopamine transporter mRNA and tyrosine hydroxylase mRNA in ventral mesencephalic neurones. Mol. Brain Res. 20, 328–334. 10.1016/0169-328x(93)90059-x 7906851

[B4] Bayer H. M. Glimcher P. W. (2005). Midbrain dopamine neurons encode a quantitative reward prediction error signal. Neuron 47, 129–141. 10.1016/j.neuron.2005.05.020 15996553 PMC1564381

[B5] Chaudhury D. Walsh J. J. Friedman A. K. Juarez B. Ku S. M. Koo J. W.. (2013). Rapid regulation of depression-related behaviours by control of midbrain dopamine neurons. Nature 493, 532–536. 10.1038/nature11713 23235832 PMC3554860

[B6] Chieng B. Azriel Y. Mohammadi S. Christie M. J. (2011). Distinct cellular properties of identified dopaminergic and GABAergic neurons in the mouse ventral tegmental area. J. Physiol. 589, 3775–3787. 10.1113/jphysiol.2011.210807 21646409 PMC3171885

[B7] Chow B. Y. Han X. Dobry A. S. Qian X. Chuong A. S. Li M.. (2010). High-performance genetically targetable optical neural silencing by light-driven proton pumps. Nature 463, 98–102. 10.1038/nature08652 20054397 PMC2939492

[B8] Chuong A. S. Miri M. L. Busskamp V. Matthews G. A. C. Acker L. C. Sorensen A. T.. (2014). Noninvasive optical inhibition with a red-shifted microbial rhodopsin. Nat. Neurosci. 17, 1123–1129. 10.1038/nn.3752 24997763 PMC4184214

[B9] Cohen J. Y. Amoroso M. W. Uchida N. (2015). Serotonergic neurons signal reward and punishment on multiple timescales. Elife 4:06346. 10.7554/eLife.06346 25714923 PMC4389268

[B10] Danjo T. Yoshimi K. Funabiki K. Yawata S. Nakanishi S. (2014). Aversive behavior induced by optogenetic inactivation of ventral tegmental area dopamine neurons is mediated by dopamine D2 receptors in the nucleus accumbens. Proc. Natl. Acad. Sci. U S A 111, 6455–6460. 10.1073/pnas.1404323111 24737889 PMC4036004

[B11] Everitt B. J. Robbins T. W. (2005). Neural systems of reinforcement for drug addiction: from actions to habits to compulsion. Nat. Neurosci. 8, 1481–1489. 10.1038/nn1579 16251991

[B12] Fenno L. Yizhar O. Deisseroth K. (2011). The development and application of optogenetics. Annu. Rev. Neurosci. 34, 389–412. 10.1146/annurev-neuro-061010-113817 21692661 PMC6699620

[B13] Gradinaru V. Zhang F. Ramakrishnan C. Mattis J. Prakash R. Diester I.. (2010). Molecular and cellular approaches for diversifying and extending optogenetics. Cell 141, 154–165. 10.1016/j.cell.2010.02.037 20303157 PMC4160532

[B14] Gunaydin L. A. Grosenick L. Finkelstein J. C. Kauvar I. V. Fenno L. E. Adhikari A.. (2014). Natural neural projection dynamics underlying social behavior. Cell 157, 1535–1551. 10.1016/j.cell.2014.05.017 24949967 PMC4123133

[B15] Han X. Chow B. Y. Zhou H. Klapoetke N. C. Chuong A. Rajimehr R.. (2011). A high-light sensitivity optical neural silencer: development and application to optogenetic control of non-human primate cortex. Front. Syst. Neurosci. 5:18. 10.3389/fnsys.2011.00018 21811444 PMC3082132

[B16] Herde M. K. Iremonger K. J. Constantin S. Herbison A. E. (2013). GnRH neurons elaborate a long-range projection with shared axonal and dendritic functions. J. Neurosci. 33, 12689–12697. 10.1523/jneurosci.0579-13.2013 23904605 PMC6618539

[B17] Ibáñez-Sandoval O. Tecuapetla F. Unal B. Shah F. Koós T. Tepper J. M. (2010). Electrophysiological and morphological characteristics and synaptic connectivity of tyrosine hydroxylase-expressing neurons in adult mouse striatum. J. Neurosci. 30, 6999–7016. 10.1523/jneurosci.5996-09.2010 20484642 PMC4447206

[B18] Kapoor V. Provost A. C. Agarwal P. Murthy V. N. (2016). Activation of raphe nuclei triggers rapid and distinct effects on parallel olfactory bulb output channels. Nat. Neurosci. 19, 271–282. 10.1038/nn.4219 26752161 PMC4948943

[B19] Kim J. H. Lee S.-R. Li L.-H. Park H.-J. Park J.-H. Lee K. Y.. (2011). High cleavage efficiency of a 2A peptide derived from porcine Teschovirus-1 in human cell lines, zebrafish and mice. PLoS One 6:e18556. 10.1371/journal.pone.0018556 21602908 PMC3084703

[B20] Kolisnyk B. Guzman M. S. Raulic S. Fan J. Magalhaes A. C. Feng G.. (2013). ChAT-ChR2-EYFP mice have enhanced motor endurance but show deficits in attention and several additional cognitive domains. J. Neurosci. 33, 10427–10438. 10.1523/JNEUROSCI.0395-13.2013 23785154 PMC6618591

[B21] Krashia P. Martini A. Nobili A. Aversa D. D’Amelio M. Berretta N.. (2017). On the properties of identified dopaminergic neurons in the mouse substantia nigra and ventral tegmental area. Eur. J. Neurosci. 45, 92–105. 10.1111/ejn.13364 27519559

[B22] Lammel S. Steinberg E. E. Földy C. Wall N. R. Beier K. Luo L.. (2015). Diversity of transgenic mouse models for selective targeting of midbrain dopamine neurons. Neuron 85, 429–438. 10.1016/j.neuron.2014.12.036 25611513 PMC5037114

[B23] Lamprecht M. R. Sabatini D. M. Carpenter A. E. (2007). CellProfiler: free, versatile software for automated biological image analysis. Biotechniques 42, 71–75. 10.2144/000112257 17269487

[B24] Latchoumane C.-F. V. Ngo H.-V. V. Born J. Shin H.-S. (2017). Thalamic spindles promote memory formation during sleep through triple phase-locking of cortical, thalamic and hippocampal rhythms. Neuron 95, 424.e6–435.e6. 10.1016/j.neuron.2017.06.025 28689981

[B25] Li P. Snyder G. L. Vanover K. E. (2016a). Dopamine targeting drugs for the treatment of schizophrenia: past, present and future. Curr. Top. Med. Chem. 16, 3385–3403. 10.2174/1568026616666160608084834 27291902 PMC5112764

[B26] Li Y. Zhong W. Wang D. Feng Q. Liu Z. Zhou J.. (2016b). Serotonin neurons in the dorsal raphe nucleus encode reward signals. Nat. Commun. 7:10503. 10.1038/ncomms10503 26818705 PMC4738365

[B27] Liu Z. Zhou J. Li Y. Hu F. Lu Y. Ma M.. (2014). Dorsal raphe neurons signal reward through 5-HT and glutamate. Neuron 81, 1360–1374. 10.1016/j.neuron.2014.02.010 24656254 PMC4411946

[B28] Lorang D. Amara S. G. Simerly R. B. (1994). Cell-type-specific expression of catecholamine transporters in the rat brain. J. Neurosci. 14, 4903–4914. 10.1523/jneurosci.14-08-04903.1994 8046459 PMC6577178

[B29] Lottem E. Lörincz M. L. Mainen Z. F. (2016). Optogenetic activation of dorsal raphe serotonin neurons rapidly inhibits spontaneous but not odor-evoked activity in olfactory cortex. J. Neurosci. 36, 7–18. 10.1523/jneurosci.3008-15.2016 26740645 PMC6601795

[B30] Mahn M. Prigge M. Ron S. Levy R. Yizhar O. (2016). Biophysical constraints of optogenetic inhibition at presynaptic terminals. Nat. Neurosci. 19, 554–556. 10.1038/nn.4266 26950004 PMC4926958

[B31] Mattis J. Tye K. M. Ferenczi E. A. Ramakrishnan C. O’Shea D. J. Prakash R.. (2011). Principles for applying optogenetic tools derived from direct comparative analysis of microbial opsins. Nat. Methods 9, 159–172. 10.1038/nmeth.1808 22179551 PMC4165888

[B32] Michelsen K. A. Schmitz C. Steinbusch H. W. M. (2007). The dorsal raphe nucleus—from silver stainings to a role in depression. Brain Res. Rev. 55, 329–342. 10.1016/j.brainresrev.2007.01.002 17316819

[B33] Miyazaki K. W. Miyazaki K. Tanaka K. F. Yamanaka A. Takahashi A. Tabuchi S.. (2014). Optogenetic activation of dorsal raphe serotonin neurons enhances patience for future rewards. Curr. Biol. 24, 2033–2040. 10.1016/j.cub.2014.07.041 25155504

[B34] Mlinar B. Montalbano A. Piszczek L. Gross C. Corradetti R. (2016). Firing properties of genetically identified dorsal raphe serotonergic neurons in brain slices. Front. Cell. Neurosci. 10:195. 10.3389/fncel.2016.00195 27536220 PMC4971071

[B35] Nelson A. B. Hammack N. Yang C. F. Shah N. M. Seal R. P. Kreitzer A. C. (2014). Striatal cholinergic interneurons drive GABA release from dopamine terminals. Neuron 82, 63–70. 10.1016/j.neuron.2014.01.023 24613418 PMC3976769

[B36] Pignatelli A. Belluzzi O. (2017). Dopaminergic neurones in the main olfactory bulb: an overview from an electrophysiological perspective. Front. Neuroanat. 11:7. 10.3389/fnana.2017.00007 28261065 PMC5306133

[B37] Ray R. S. Corcoran A. E. Brust R. D. Kim J. C. Richerson G. B. Nattie E.. (2011). Impaired respiratory and body temperature control upon acute serotonergic neuron inhibition. Science 333, 637–642. 10.1126/science.1205295 21798952 PMC3729433

[B38] Redgrave P. Rodriguez M. Smith Y. Rodriguez-Oroz M. C. Lehericy S. Bergman H.. (2010). Goal-directed and habitual control in the basal ganglia: implications for Parkinson’s disease. Nat. Rev. Neurosci. 11, 760–772. 10.1038/nrn2915 20944662 PMC3124757

[B39] Schindelin J. Arganda-Carreras I. Frise E. Kaynig V. Longair M. Pietzsch T.. (2012). Fiji: an open-source platform for biological-image analysis. Nat. Methods 9, 676–682. 10.1038/nmeth.2019 22743772 PMC3855844

[B40] Schultz W. Dayan P. Montague P. R. (1997). A neural substrate of prediction and reward. Science 275, 1593–1599. 10.1126/science.275.5306.1593 9054347

[B41] Steinberg E. E. Keiflin R. Boivin J. R. Witten I. B. Deisseroth K. Janak P. H. (2013). A causal link between prediction errors, dopamine neurons and learning. Nat. Neurosci. 16, 966–973. 10.1038/nn.3413 23708143 PMC3705924

[B42] Tan K. R. Yvon C. Turiault M. Mirzabekov J. J. Doehner J. Labouèbe G.. (2012). GABA neurons of the VTA drive conditioned place aversion. Neuron 73, 1173–1183. 10.1016/j.neuron.2012.02.015 22445344 PMC6690362

[B43] Ting J. T. Feng G. (2014). Recombineering strategies for developing next generation BAC transgenic tools for optogenetics and beyond. Front. Behav. Neurosci. 8:111. 10.3389/fnbeh.2014.00111 24772073 PMC3982106

[B44] Tye K. M. Mirzabekov J. J. Warden M. R. Ferenczi E. A. Tsai H.-C. Finkelstein J.. (2013). Dopamine neurons modulate neural encoding and expression of depression-related behaviour. Nature 493, 537–541. 10.1038/nature11740 23235822 PMC4160519

[B45] Van Dort C. J. Zachs D. P. Kenny J. D. Zheng S. Goldblum R. R. Gelwan N. A.. (2015). Optogenetic activation of cholinergic neurons in the PPT or LDT induces REM sleep. Proc. Natl. Acad. Sci. U S A 112, 584–589. 10.1073/pnas.1423136112 25548191 PMC4299243

[B46] Walther D. J. Peter J.-U. Bashammakh S. Hortnagl H. Voits M. Fink H.. (2003). Synthesis of serotonin by a second tryptophan hydroxylase isoform. Science 299:76. 10.1126/science.1078197 12511643

[B47] Wiegert J. S. Mahn M. Prigge M. Printz Y. Yizhar O. (2017). Silencing neurons: tools, applications and experimental constraints. Neuron 95, 504–529. 10.1016/j.neuron.2017.06.050 28772120 PMC5830081

[B48] Wise R. A. (2004). Dopamine, learning and motivation. Nat. Rev. Neurosci. 5, 483–494. 10.1038/nrn1406 15152198

[B49] Xenias H. S. Ibáñez-Sandoval O. Koós T. Tepper J. M. (2015). Are striatal tyrosine hydroxylase interneurons dopaminergic? J. Neurosci. 35, 6584–6599. 10.1523/jneurosci.0195-15.2015 25904808 PMC4405564

[B50] Yohn C. N. Gergues M. M. Samuels B. A. (2017). The role of 5-HT receptors in depression. Mol. Brain 10:28. 10.1186/s13041-017-0306-y 28646910 PMC5483313

[B51] Zhang H. Zhao H. Zeng C. Van Dort C. Faingold C. L. Taylor N. E.. (2018). Optogenetic activation of 5-HT neurons in the dorsal raphe suppresses seizure-induced respiratory arrest and produces anticonvulsant effect in the DBA/1 mouse SUDEP model. Neurobiol. Dis. 110, 47–58. 10.1016/j.nbd.2017.11.003 29141182 PMC5748009

[B52] Zhao S. Ting J. T. Atallah H. E. Qiu L. Tan J. Gloss B.. (2011). Cell type-specific channelrhodopsin-2 transgenic mice for optogenetic dissection of neural circuitry function. Nat. Methods 8, 745–752. 10.1038/nmeth.1668 21985008 PMC3191888

