# Supplementary material for: Two eARCHT3.0 Lines for Optogenetic Silencing of Dopaminergic and Serotonergic Neurons
Source: Front Neural Circuits. 2019 Feb 1;13:4. doi: 10.3389/fncir.2019.00004 (PMC6367884; doi:10.3389/fncir.2019.00004)

Supplementary Material

Two eARCHT3.0 lines for optogenetic silencing of dopaminergic and serotonergic neurons.

Alexandra Krol[1^], Violeta G. Lopez-Huerta[1,2^], Taylor E. C. Corey[1], Karl Deisseroth[3,4,5], Jonathan T. Ting[1,6*], Guoping Feng[1,2*].

*** Correspondence:** Corresponding Authors:
JonathanT@alleninstitute.org, fengg@mit.edu

Supplemental Figure 1 **Fiber placement**

The positions of the ends of the optic fiber tracks are schematized for each animal analyzed for conditional place aversion, reconstructed from serial sections

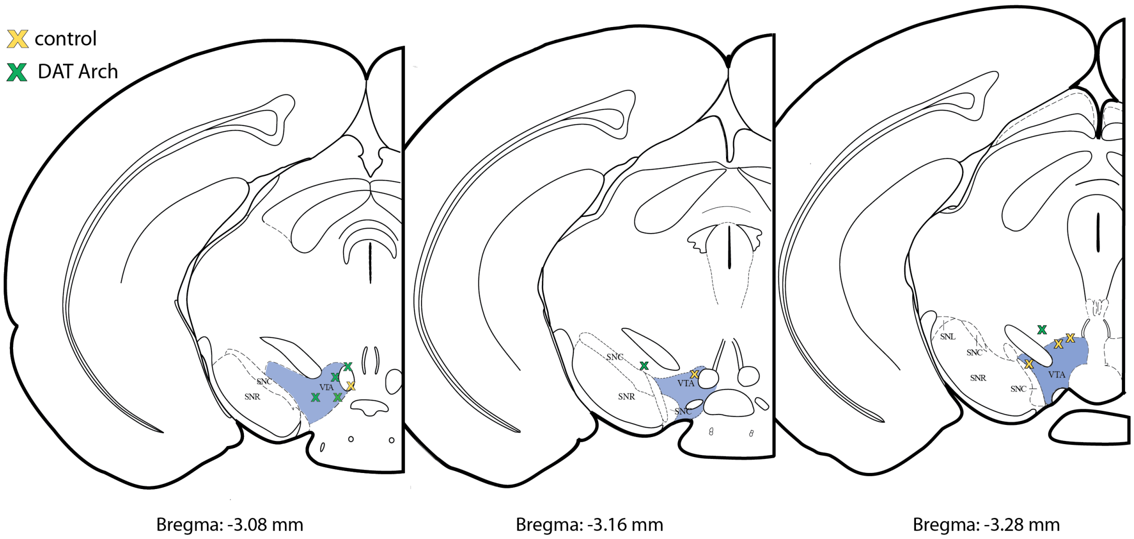

Supplementary Figure 2 **GFP expression closely mirrors DAT expression**

(**A)** Sagittal section, merged image of B-D. (**B)** Expression of eArchT3.0, marked by GFP fluorescence (**C)** TH immunostaining. (**D)** DAT immunostaining. Scale bar 1 mm.

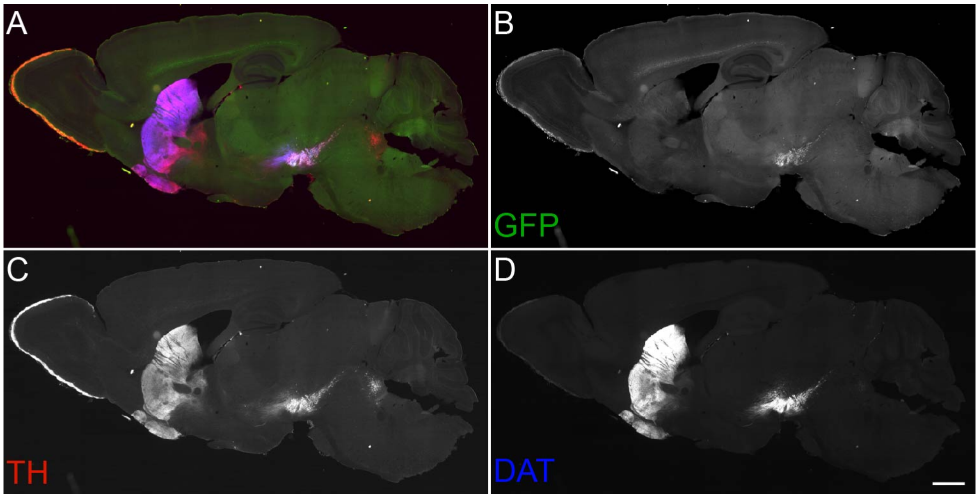

Supplementary Figure 3 **Additional GFP expression in DAT- eArchT3.0 line**

(A) Medial sagittal section with GFP and TH staining. Scale bar 1 mm. Inset (B,C) is of dorsal raphe. (**B)** GFP. **(C)** TH positive neurons. Scale bar 0.5 mm. (**D)** Lateral sagittal section with GFP fluorescence. Scale bar 1mm. Insets (E,F). (**E)** Cortex. Off-target GFP expression in scattered cortical neurons. Scale bar 100um. **(F)** Cortical subplate and striatum. Off-target GFP expression in subplate neurons. On-target GFP expression in small striatal interneurons which are TH positive (not shown). Scale bar 100um.

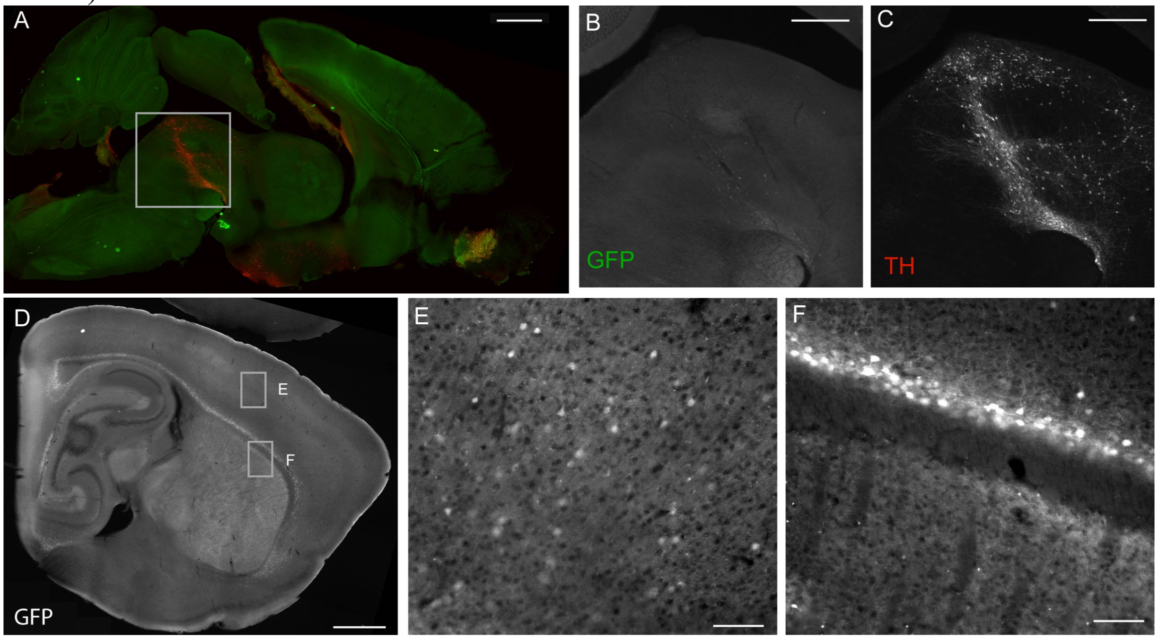

Supplementary Figure 4 **Lack of long-term effects of BAC eArchT3.0 transgene in DAT and TPH2-eArchT3.0 transgenic mice.**

(A-C) Example 20x confocal images of sagittal sections used for quantification. Scale bars are 100um. **(A)** TH staining of the VTA/SNc. **(B)** DAT staining of the striatum. **(C)** TPH2 staining of the DRN. **(D)** Number TH+ cells in the VTA/SNc per animal summed across 3 sections per animals, (Control, 689±201, DAT-eArchT3.0 793±189, n=3 animals). **(E)** Median DAT intensity in striatum. (Control 0.014±.006, DAT-eArchT3.0 0.015±.004, n=12 sections from 3 animals). **(F)** Number TPH2+ cells in the DRN per animal summed across 3 sections. (Control 1032±159, TPH2-eArchT3.0 728±224, n=3 animals). **(G)** Median TPH2 intensity in the DRN, normalized for cell area coverage per section from cells identified in (F). (Control 0.006±.005, TPH2-eArchT3.0 0.004±.004, n=9 sections from 3 animals). (Quantification: **(**D-G**)** mean ± SD, n.s. unpaired t-test).

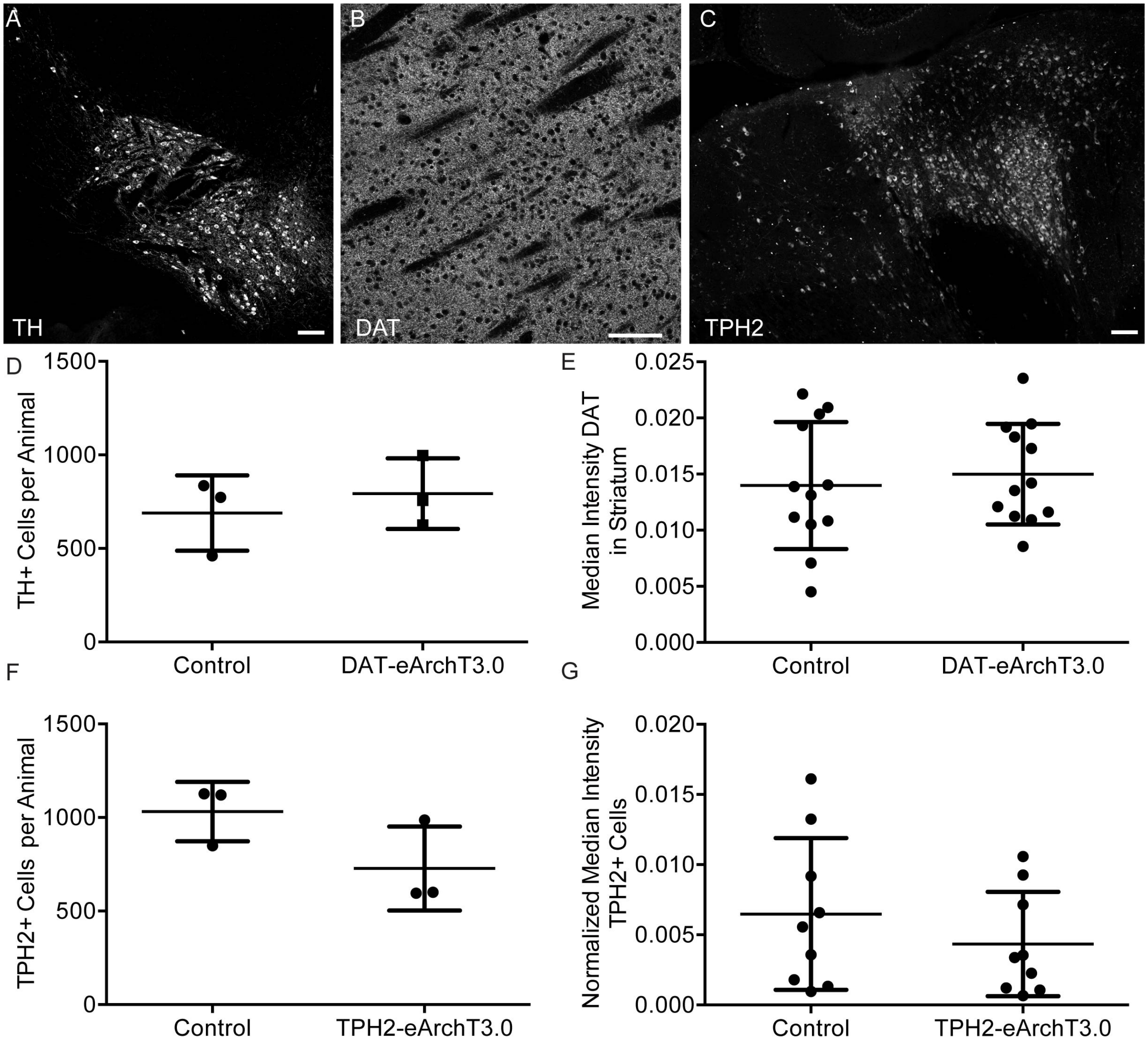

Supplementary Figure 5 **Additional GFP expression in TPH2-eArchT3.0 line**

(**A)** Lateral sagittal section, GFP expression. Scale bar 1mm. Insets: **(A')** Cerebellum. **(A'')** Dorsal Raphe. **(A''')** Cortex. **(A'''')** Olfactory bulb. Scale bar 100um. **(B)** Medial sagittal section, GFP expression. Scale bar 1mm. Insets: **(B')** Cerebellum. **(B'')** Dorsal Raphe. **(B''')** Cortex. **(B'''')** Olfactory bulb. Scale bar 100um.

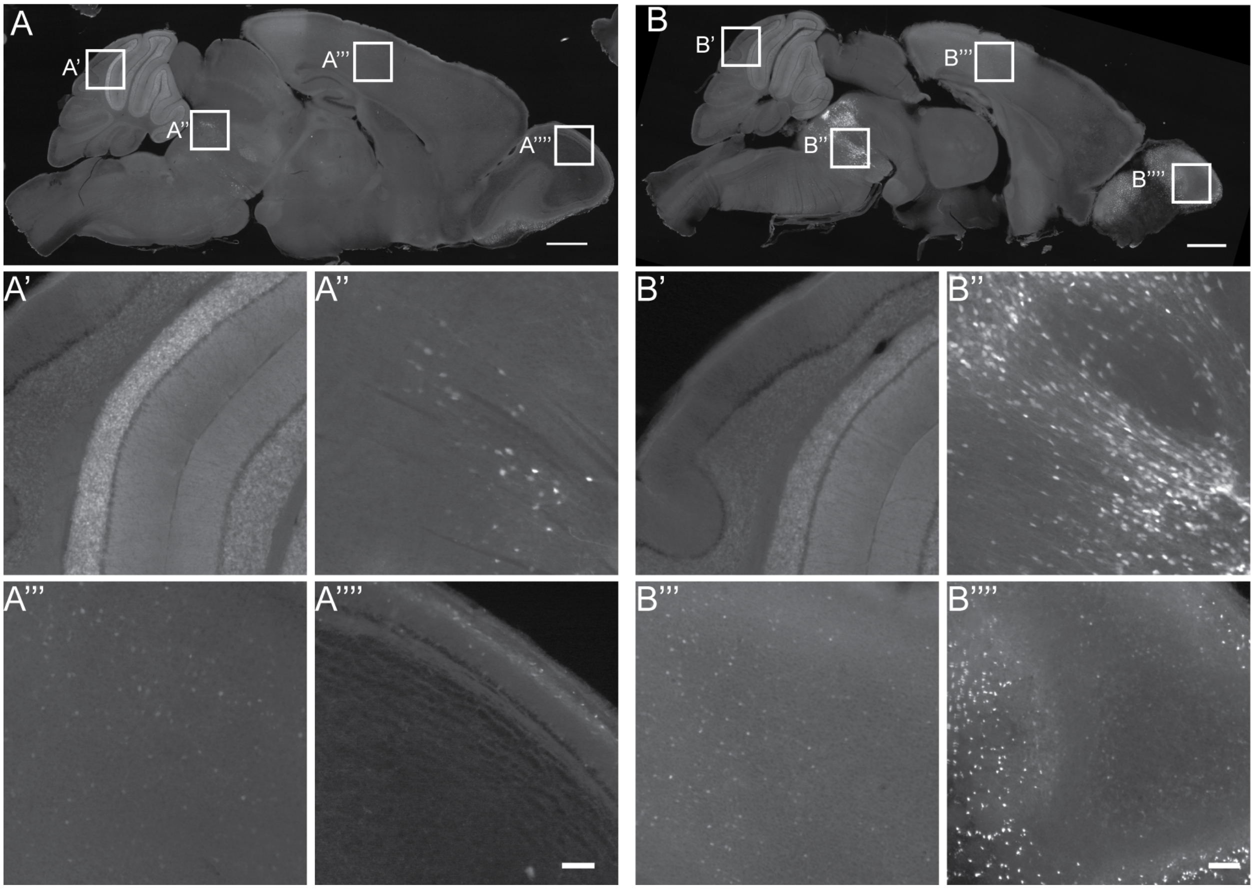

Supplementary Figure 6 **Electrophysiological characterization of eArchT3.0 GFP negative neurons in DAT-eArchT3.0 and TPH2-eArchT3.0 transgenic mice.**

(A) Schematic representation of the recording and stimulation preparation. **(B)** VTA neurons' response to hyperpolarizing and depolarizing current steps. We found two types of GFP (-) neurons in the VTA. **(C)** Population summary of changes evoked by photo stimulation in the resting membrane potential (RMP) (light OFF -52.4±2.2 mV and light ON -53.2±2.1 mV, n.s. unpaired t-test, n=9, mean ± SEM) or the spike frequency (light OFF 5.9±2 Hz and light ON 6.1±2.2, n.s. unpaired t-test, n=5, mean ± SEM) (left). In the right panels, examples of the response to photo stimulation in both cell types, voltage clamp traces (top) and current clamp traces (bottom) with and without photo stimulation (green bar, 532 nm light). **(D)** Schematic representation of the recording and stimulation preparation. **(E)** DRN GFP (-) neurons' response to hyperpolarizing and depolarizing current steps. **(F)** Population summary of the changes in the spike frequency evoked by photo stimulation (left, light OFF 0.85±0.26 Hz and light ON 0.81±0.35 Hz, n.s. unpaired t-test, n=8, mean ± SEM). In the right panel, examples of the response to photo stimulation, voltage clamp traces (top) and current clamp traces (bottom) with and without photo stimulation (green bar, 532 nm light).

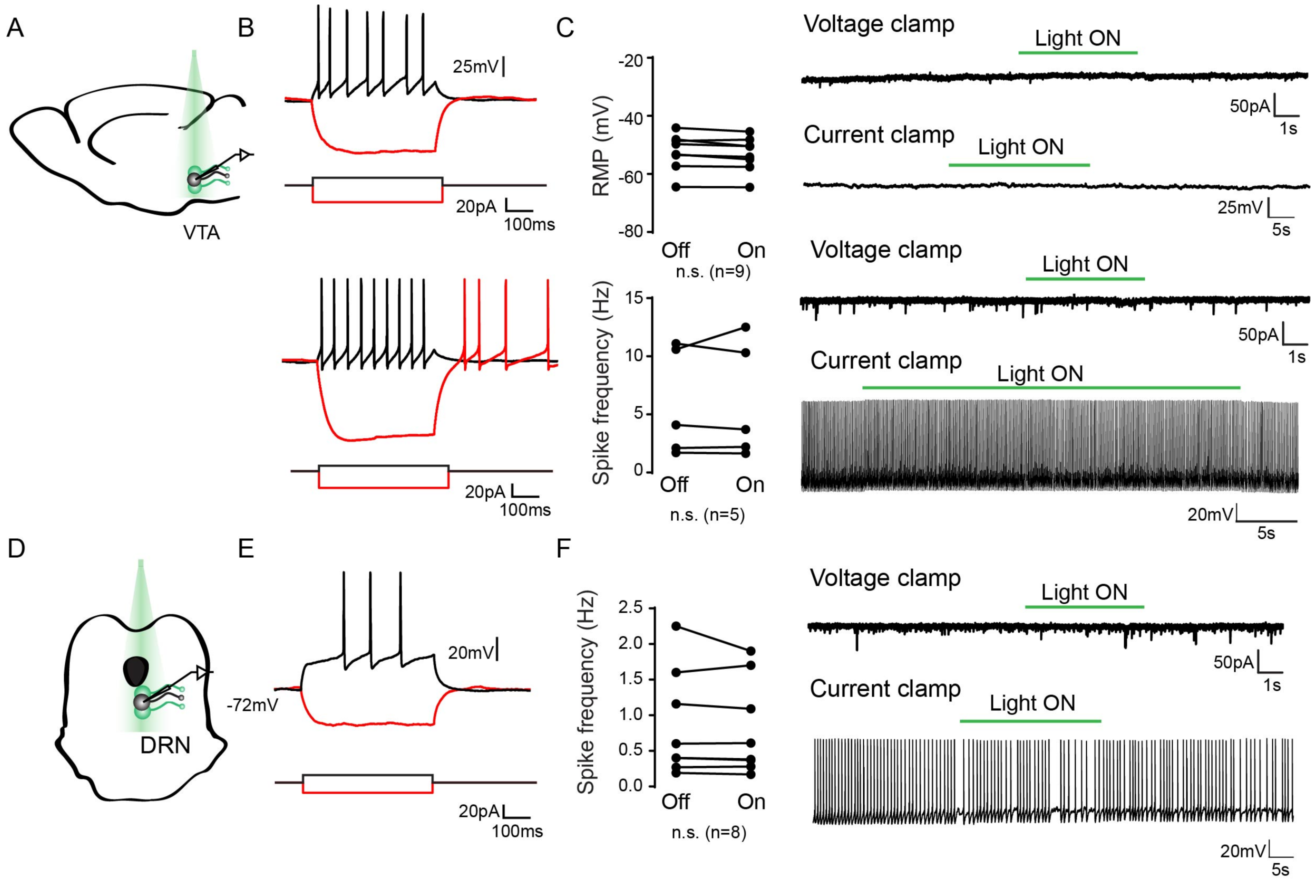

Supplement: Supplementary file 1 [file Data_Sheet_1.pdf]
